# A Conserved Secondary Structural Element in the Coding Region of the Influenza A Virus Nucleoprotein (NP) mRNA Is Important for the Regulation of Viral Proliferation

**DOI:** 10.1371/journal.pone.0141132

**Published:** 2015-10-21

**Authors:** Marta Soszynska-Jozwiak, Paula Michalak, Walter N. Moss, Ryszard Kierzek, Elzbieta Kierzek

**Affiliations:** 1 Institute of Bioorganic Chemistry, Polish Academy of Sciences, 61–704 Poznan, Noskowskiego 12/14, Poland; 2 Department of Molecular Biophysics and Biochemistry, Howard Hughes Medical Institute, Yale University School of Medicine, New Haven, Connecticut, United States of America; University of British Columbia, CANADA

## Abstract

Influenza A virus is a threat to humans due to seasonal epidemics and infrequent, but dangerous, pandemics that lead to widespread infection and death. Eight segments of RNA constitute the genome of this virus and they encode greater than eight proteins via alternative splicing of coding (+)RNAs generated from the genomic (-)RNA template strand. RNA is essential in its life cycle. A bioinformatics analysis of segment 5, which encodes nucleoprotein, revealed a conserved structural motif in the (+)RNA. The secondary structure proposed by energy minimization and comparative analysis agrees with structure predicted based on experimental data using a 121 nucleotide *in vitro* RNA construct comprising an influenza A virus consensus sequence and also an entire segment 5 (+)RNA (strain A/VietNam/1203/2004 (H5N1)). The conserved motif consists of three hairpins with one being especially thermodynamically stable. The biological importance of this conserved secondary structure is supported in experiments using antisense oligonucleotides in cell line, which found that disruption of this motif led to inhibition of viral fitness. These results suggest that this conserved motif in the segment 5 (+)RNA might be a candidate for oligonucleotide-based antiviral therapy.

## Introduction

Influenza A virus is a grave threat to human health. In 1918 the Spanish flu (H1N1strain) caused the deaths of over 50 million people [1, 2]. In 1997, migration of the H5N1 strain from bird to human was verified by molecular analysis [3]. In 2007, a new pandemic strain of influenza virus H1N1 was observed. This is particularly virulent and can rapidly disseminate itself through humans [4]. New pandemic strains of influenza virus are likely to arise in the 21st century, which makes the investigation of novel therapeutic targets in influenza especially relevant.

Influenza A virus belongs to the family *Orthomyxoviridae* and possesses a segmented, negative-sense RNA genomic viral (v)RNA. RNA is used throughout infection and plays roles in every process of viral life cycle. Replication begins with vRNA acting as a template to produce two plus-sense (+)RNAs: a complementary (c)RNA intermediate, which in turn becomes the template for producing more vRNA strands, as well as protein coding mRNA [5, 6]. A number of publications have suggested that RNA secondary structure plays important roles in influenza A infection [7–15]. A bioinformatics analysis based on identifying regions of unusual thermodynamic stability and structural conservation revealed that the (+) sense influenza RNA contains at least twelve structured motifs with likely function [12, 15]. Several of these regions overlapped areas of suppressed synonymous codon usage [12, 16], which suggests that RNA structure is exerting an evolutionary constraint on influenza A virus codon evolution.

The best-studied structured regions occur in the mRNAs of segments 7 and 8, where structures are modeled at or near splice sites. Indeed, predicted structural regions appear at or near splice sites in influenza B and C, suggesting common roles for structure in the regulation of influenza alternative splicing [16]. In influenza A, for example, a 63 nucleotide (nt) conserved region was identified in the segment 7 mRNA. This region can fold in two conformations: a hairpin and pseudoknot [11]. Changing between these two conformations places splicing regulatory elements into varying structural contexts, which has likely implications on segment 7 splicing. Another conserved structure occurs in the intron of segment 8 mRNA [14]. The function of this domain is unclear, but its proximity to the 5’ splice site makes it a possible intronic splicing enhancer/inhibitor. Also the defined structure could be a tag for recognition of proteins to distinguish unspliced NS1 mRNA from spliced NEP mRNA. Another conserved motif, in influenza mRNA of segment 7 was determined biochemically [13]. A multibranch loop structure is proposed to modulate alternative splicing of segment 7; as deduced by comparing previous point mutations studies [17] to the model [13]. Understanding the roles of influenza RNA thus plays an important role in gaining mechanistic insights into influenza virology and, significantly, in designing new drugs that can target viral RNA/RNA structure.

A previous bionformatic analysis of six (+)RNA sequences from segment 5 predicted a high probability of structure in the region spanning nts 1031–1250 [12]. This regions has unusually stable predicted thermodynamic stability and the model base pairs are conserved between homologous sequences. Additionally, the structure in this region appears to be influencing amino acid codon evolution, where synonymous codon use is highly suppressed (vs. other regions of influenza); presumably due to the need of maintaining structure in base-paired third codon (wobble) sites. In this study, we focused on a structured region of influenza A segment 5. This segment encodes nucleoprotein (NP), which is a structural protein and also takes part in regulation of transcription and replication. NP with viral polymerase and vRNAs create eight ribonucleoprotein complexes (RNP) which determine virus particle structure. Here, a new predicted motif in (+) RNA of segment 5 (nts 1051 to 1171) was confirmed *in vitro* and in cell line experiments. Chemical and enzymatic probing, Pb^2+^ cleavage and isoenergetic microarray mapping were used to probe the secondary structure a truncated *in vitro* construct comprising the consensus sequence of this NP mRNA motif. This isolated structure was also confirmed by probing the entire segment 5 (+)RNA of strain A/VietNam/1203/2004 (H5N1). Antisense oligonucleotides targeting a hairpin in this conserved motif are able inhibit virus proliferation in cell line, suggesting functional significance for this structure.

## Materials and Methods

### Oligonucleotides synthesis

Primers for PCR and reverse transcription and microarrays probes and antisense oligonucleotides were synthesized on MerMade12 (BioAutomation) synthesizer, using β-cyanoethyl phosphoramidite chemistry on solid support. Oligonucleotides were deprotected and purified according to published protocol [18, 19]. Concentrations of all oligonucleotides were measured using a Spectrophotometer UV (Picodrop-Syngen).

### Experimental Constructs

DNA templates for synthesis of M121 was obtained from PCRs reactions using appropriate primers (Table 1). Two partially complementary DNA oligonucleotides S5-1 and S5-2 and were used in first PCR reaction. The product from the PCR was temple for second PCR reaction with primers contains EcoRI and PstI restriction sites: M-F and M-R. The PCR final product was ligated into a digested pUC19 vector. The resulting plasmid was cloned into DH5-alpha cells. Plasmid was isolated from cloned cells using a Qiagen mini-prep kit and the sequence was confirmed by sequencing.

**Table 1:** Primers for PCR and transcription of M121 and (+)RNA5.

name	sequence
S5-1	GCGTAATACGACTCACTATAGGAAGCTTCATCAGAGGGACAAGAGTGGTCCCAAGAGGACAACTGTCCACCAGAGGAGTTCA
S5-2	TCTGCTTCTCAGTTCAAGAGTACTGGAGTCCATTGTTTCCATGTTCTCATTTGAAGCAATTTGAACTCCTCTGGTGGACAG
M_F	CAC*GAATTC*GCGTAATACGACTCACTATAGGAAGC
M_R	CGC*GACGTC*TCTGCTTCTCAGTTCAAGAGTACTG
T1-F	TCTGCTTCTCAGTTCAAGAGTACTG
T2-R	GCGTAATACGACTCACTATAGGAAGC
Pr1 (M121 RT primer)	TCTGCTTCTCAGTTCAAGAGTAC
For_c5	GCGTAATACGACTCACTATAGGGAGCAAAAGCAGGGTAGATAATC
Rev_c5	AGTAGAAACAAGGGTATTTTTC
Pr2 ((+)RNA5 RT primer) region 1302–1324	CCATAATGGTCGCTCTTTCGAAG

### M121 RNA and (+)RNA5 synthesis

PCR reaction with specific primers T1-F and T2-R on above digested plasmid was perform to produce templates for transcription. *In vitro* transcription reactions was performed using an Ampliscribe T7 Flash kit (Epicenter). Product was purified by denaturing 8% PAGE gel and eluted. M121 RNA for folding experiment, enzymatic mapping and lead ion cleavage was 5’ end labeled with γ-32P ATP (Hartmann Analityc GmbH), then re-purified by denaturing PAGE.

DNA template for *in vitro* transcriptions of (+)RNA5 (1565 nt) was obtained by PCR from vector pPol1 using primers For_c5 and Rev_c5 (Table 1). The pPol1 vector containing DNA of segment 5 influenza strain A/Viet Nam/1203/2004 (H5N1) was received from Prof. Baek Kim, University of Rochester. (+)RNA5 was purified using RNeasy MiniElute Cleanup kit (Qiagen) and quality of RNA was checked on agarose gel.

### M121 RNA and (+)RNA5 Folding

For each sample, about 20,000 cpm of 5' labeled M121 RNA in folding buffer (100 mM Tris-HCl pH 7, 100 mM KCl and different concentration of MgCl_2_) was heated in 65°C for 5 min and slowly cooled to room temperature for 50 min. To study the multivalent cation dependent folding, MgCl_2_ was used to get a range of final concentration from 2.5 mM to 25 mM. Also Co^3+^ dependence folding was studied using Co(NH_3_)_6_Cl_3_ added to span 0.002 to 10 mM. Folding was analyzed by native gel electrophoresis using 6% polyacrylamide gel with 1x THEM (34 mM Tris Base, 57 mM HEPES, 0.1 mM EDTA, 10 mM MgCl_2_) buffer running at 4°C with low voltage. In all conditions one band was achieved. For next experiments the folding buffer contained 100 mM KCl and 5 mM MgCl_2_ was chosen. Entire (+)RNA5 was folded in the same condition and obtaining of one structure was checked on agarose gel in native conditions (running at 4°C).

### Chemical and Enzymatic Mapping and lead ion cleavage

M121 and (+)RNA5 for SHAPE (Selective 2′-Hydroxyl Acylation analyzed by Primer Extension) and chemical mapping experiments were folded in buffer 100 mM KCl, 5 mM MgCl_2_, 50 mM HEPES pH 7.0. Enzymatic mapping and lead cleavage used 10 mM Tris-HCl pH 7.0 instead of 50 mM HEPES pH 7.0 and were done only for M121 RNA. Enzymatic and chemical mapping was carried out at room temperature.

Conditions for RNase S1, V1, T1 cleavages and RNase T1 ladder were adapted from manufacturer's protocol (Ambion). Optimal enzymatic concentrations were determined with enzymes titration. Lead ion cleavage reactions were carried out by incubating 5'-end labeled M121 RNA with 1 mM Pb(OAc)_2_. Aliquots with RNA (20,000 cpm per aliquot) were removed at 0, 1, 5, 15, 30, 60 min. The enzymatic or hydrolysis reactions were stopped by placing the aliquots in the gel loading buffer and freezing at -80°C until they were fractionated on a denaturing, 12% polyacrylamide gel. All gel were dried, exposed to phosphorimager screen, imaged and analyzed in MultiGauge program.

M121 RNA and (+)RNA5 were folded in folding buffer (100 mM KCl, 5 mM MgCl_2_, 50 mM HEPES, pH 7.0) as described above. Next, RNA was modified with optimized concentrations of DMS (dimethyl sulfate), CMCT (1-cyclohexyl-(2-morpholinoethyl) carbodiimide metho-p-toluene sulfonate), kethoxal and NMIA (N-methylisatoic anhydride) using slightly modified published protocols [20, 21]. For single reaction 1 pmol of RNA was used. Chemical mapping was carried out at room temperature with DMS, CMCT, kethoxal or NMIA for 15, 20, 30 or 40 min, respectively. Control reaction was done in the same conditions but without reagents. Modifications were read out by primer extension using primers listed in Table 2, with reverse transcriptase SuperScript III and Invitrogen protocols. RNA fragments and ddNTP ladders were separated by capillary electrophoresis.

**Table 2:** Isoenergetic microarrays probes that bind strongly and moderately to M121 and their thermodynamic properties.

Binding sites for M121^a^	Probe sequence^b^	Strength of probe binding^c^			ΔG°_37_ of duplex for complementary binding site^d^ (kcal/mol)	ΔG°_37_ of duplex for possible mismatched sites^e^ (kcal/mol)
		4°C	23°C	37°C		
1055 [1117]	GD^L^AG^L^CG^L^	W	W	M	-9.54	
1056	U^L^GD^L^DG^L^G^L^	W	W	-	-12.1	-5.1 (1118/1119)
1059	U^L^GD^L^UG^L^G^L^	M	M	-	-9.04	
1060	CU^L^GA^L^UG^L^	M	W	-	-9.43	
1062	CUC^L^UG^L^G^L^	S	S	M	-9.14	-9.7 (1100/1101)
1063 [1102]	CC^L^UC^L^UG^L^	S	S	S	-12.03 -10.09	-9.3 (1101/1102) -7.5 (1084)
1065	UC^L^CC^L^UG^L^	W	W	-	-10.47	-7.7 (1056)
1072	DC^L^UC^L^UG^L^	S	S	S	-9.12	-6.5 (1101) -6.3 (1061/1062) -4.5 (1105)
1073	CD^L^CU^L^CG^L^	M	M	M	-8.68	
1081	C^L^UU^L^GG^L^G^L^	W	W	-	-12.05	
1084	CC^L^UC^L^UG^L^	S	S	S	-10.09	-9.3 (1062/1063) -9.3 (1101/1102)
1085	U^L^CC^L^UC^L^G^L^	M	M	-	-9.99	-8.1 (1103)
1094	GGD^L^CDG^L^	M	W	-	-10.65	
1096	GUG^L^GD^L^G^L^	M	M	M	-10.39	-4.1 (1056/1057)
1099	C^L^UG^L^GU^L^G^L^	M	M	M	-12.38	-6.3 (1059/1060)
1100	UC^L^UG^L^GG^L^	W	W	-	-9.91	-6.1 (1080/1081)
1101	CUC^L^UG^L^G^L^	S	S	M	-12.07	-7.4 (1062)
1102 [1063]	CC^L^UC^L^UG^L^	S	S	S	-10.09 -12.03	-9.3 (1101/1102) -7.5 (1084)
1103	U^L^CC^L^UC^L^G^L^	M	M	-	-9.99	-8.1 (1103) -7.8 (1085)
1105	DC^L^UC^L^CG^L^	S	S	M	-10.04	
1117 [1055]	GD^L^AG^L^CG^L^	W	W	M	-9.54	
1118	U^L^GA^L^DG^L^G^L^	M	W	-	-8.24	-4.9 (1056)
1126	U^L^UC^L^UC^L^G^L^	S	M	M	-8.12	-5.7 (1164) -5.2 (1103) -4.9 (1085)
1154	D^L^DG^L^DG^L^G^L^	M	S	M	-10.03	
1155	C^L^DD^L^GD^L^G^L^	M	S	S	-9.49	
1156	U^L^CD^L^DG^L^G^L^	W	M	-	-8.68	
1164	U^L^UC^L^UC^L^G^L^	M	M	M	-8.12	-5.4 (1126) -5.2 (1103) -4.9 (1085)
1166	G^L^CU^L^UC^L^G^L^	M	W	W	-11.62	-4.6 (1105)

^*a*^ –binding sites of probes are denoted by the middle nucleotide of the complementary target RNA region, square brackets indicate alternative complementary site of M121 with the same sequence;

^*b*^- nucleotides in capital letter (A, C, G, U, D) are 2’-O-methyl-RNA nucleotides, nucleotides with L superscript (A^L^, C^L^, G^L^, U^L^, D^L^) are locked nucleic acid (LNA) nucleotides, D and D^L^ are 2,6 –diaminopurine riboside (2’-O-methyl type or LNA, respectively);

^*c*^– Binding was considered strong (S), medium (M) and weak (W), when the integrated intensities were ≥1/3, ≥ 1/9 and ≥ 1/27 of the strongest intensity, no binding was marked as (-). Hybridization condition: buffer: 300 mM KCl, 5 mM MgCl_2_, 50 mM HEPES, pH 7.0;

^*d*^- ΔG°_37_ calculated as modified probe/RNA duplex [36];

^*e*^- calculated in RNAstructure program as RNA/RNA duplex and, in parenthesis, the site of binding for which calculation was done.

Only alternative binding sites of probes with thermodynamic stability more favorable than -4.0 kcal/mol are noted. Binding sites of probes are denoted by the middle nucleotide of the complementary RNA region (or two nucleotides for region with an even number of nucleotides).

Data were analyzed by PeakScanner. Quantitative NMIA, DMS, CMCT and kethoxal reactivaties for individual datasets were normalized to a scale in which 0 indicates an unreactive site and the average intensity at highly reactive sites is set to 1.0. The normalization factor for each datasets was determined by first excluding the most reactive 2% of peak intensities and then calculating the average for the next 8% of peak intensities. All reactivities were then divided by this average.

### Hybridization to Isoenergetic Microarrays

Isoenergetic microarrays were prepared and microarrays mapping was performed similarly to published procedures [22–24]. About 200,000 cpm of labeled RNA was folded as described above before hybridization to isoenergetic microarrays. Probes of universal microarray was spotted in triplicate. Spotting buffer, monomer U, and pentamer UUUUU (2’-O-methyl-RNA), which should show no binding to RNA, were also printed on the microarray as internal negative controls. Hybridizations were carried out in folding buffer for 18 h at 4, 23 and 37°C. Microarrays were washed for 1 min at the same temperature as hybridization occurred and then dried by centrifugation. Hybridization was visualized by exposure to a phosphorimager screen and quantitative analysis was performed with ArrayGaugeV2.1. Binding was considered strong, medium and weak, when the integrated spot intensity was ≥ 1/3, ≥ 1/9, or ≥ 1/27 of the strongest integrated spot intensity, respectively. Alternative binding sites were predicted using RNA/RNA thermodynamics.

### Sequence Alignment and Analysis

All available influenza A sequences for segment 5 were acquired from the NCBI influenza virus resource [25]. Sequences were filtered to remove identical sequences and leave a set of unique RNAs. From this set of 2515 sequences subsets of 100 were randomly selected for analysis with the program RNAz [26, 27] which scans for RNA structure using overlapping prediction windows of 120 nt in 10 nt steps. Overlapping windows with high propensity of forming functional RNA structure were concatenated and that region of the alignment was extracted for initial *in silico* modeling with the program RNAalifold [28] that predicts consensus folds for fixed alignments. Model structures were then evaluated for conservation with respect to the full alignment of all unique influenza A sequences.

### Cells experiments

Cell line experiments were performed using single-cycle infectious influenza A virus (sciIAV) of strain A/California/04_NYICE_E3/2009 (H1N1) in MDCK-HA (Madin–Darby canine kidney epithelial cells-Hemagglutinin) cells, both were gift from Prof. Luis Martinez-Sobrido, University of Rochester [29, 30]. Cells were grown at 37°C with 5% CO_2_ in Dulbecco's modified Eagle's medium (DMEM) supplemented with 10% fetal bovine serum (FBS) and penicillin-streptomycin-glutamine. Every third passage hygromycin B was added to constitutive express hemagglutinin. 96-Well plates were seeded with MDCK-HA cells (10,000 cells per well) 24 h before transfection. Lipofectamine 2000 was used to transfect 4 uM antisense oligonucleotides and 12 h after transfection, medium was changed and cells were incubated for 6 h at 37°C. Next, infection with low MOI (0.001) was performed. Post infection takes 40 h at 33°C (two viral life cycle) and all supernatants were collected for analysis. Next, serial dilutions of cell culture supernatant were used for infections of MDCK-HA monolayers in 96-well plates. Post infection time was 20 h at 33°C (one viral life cycle); this unable counting GFP foci and calculation of FFU/ml. Controls were infected cells in medium, infected cells treated with lipofectamine 2000 without any antisense oligonucleotide and infected cells transfected with 2’-O-methyl-RNA oligonucleotide AGACCUCUAUAGCAGCU (N) as negative controls [31]. Ribavirin at concentration 40 uM added in postinfection media served as positive control.

## Results and Discussion

### Identification of a conserved structural motif in segment 5 (+)RNA

**Fig 1:**
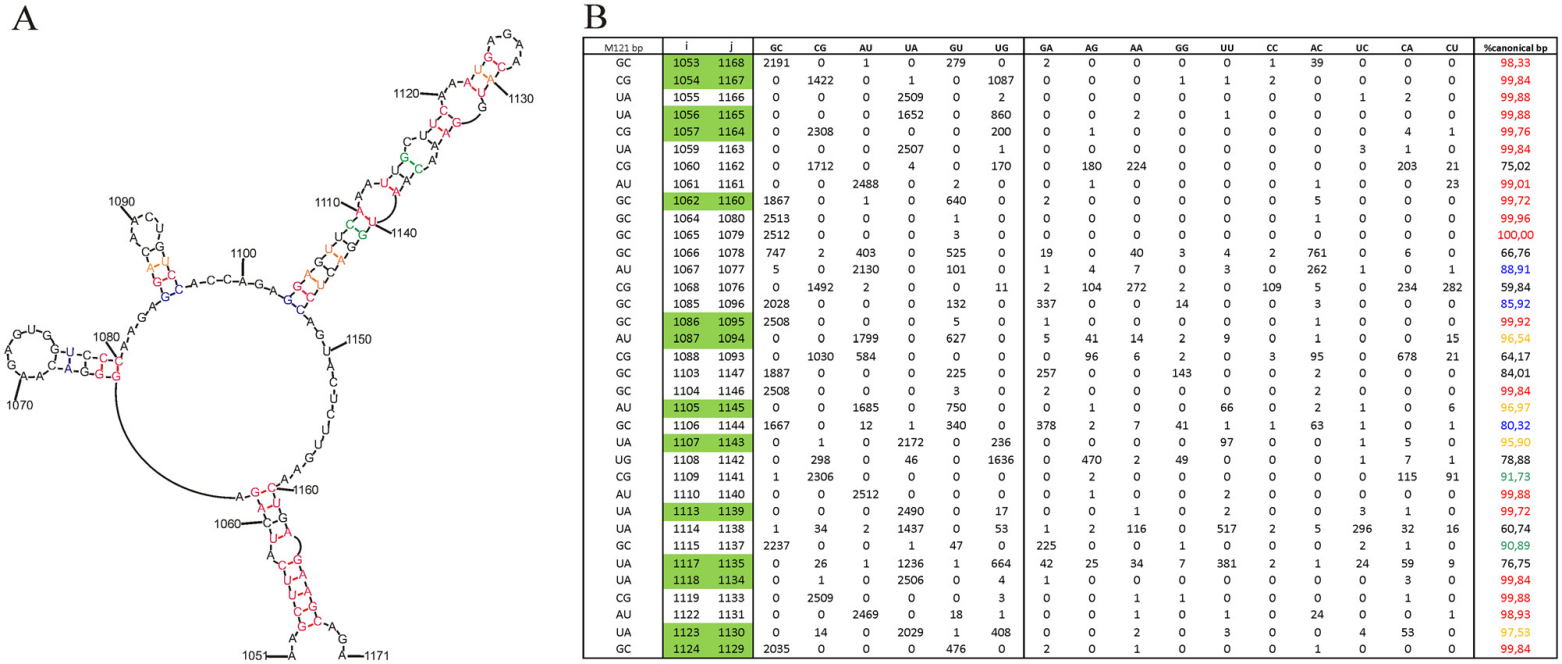
Secondary structure of M121 predicted by RNAalifold and bioinformatics calculations for possibility of each base pair. **A.** Base pairs in secondary structure are colored according to % of canonical base pairs calculated for type A influenza (last column of table): red >98%; 95 ≤ orange < 98%; 90 ≤ green < 95%; 85 ≤ blue < 90%. **B.** In table are marked preserving mutations: indicated by green shadow are evidence of compensatory mutation.

Here, we reanalyzed the segment 5 RNA using a larger set of 100 sequences with the ncRNA discovery program RNAz [26, 32]. This new analysis revealed one predicted region with a high propensity of forming structured RNA: support vector machine class probability (p-class) 0.90 and z-score of -2.49. The high p-class indicates that this region has similar calculated metrics (e.g. stability and structure conservation) as known structured ncRNAs [26, 32], and the z-score indicates that, compared to random sequence, this region is ~2.5 standard deviations more thermodynamically stable. Furthermore, compared to previous work on segment 5, the identified structured region is narrowed to nts 1051–1171, whereas the previously identified region spanned nt 1031–1250 [12]. The program RNAalifold [28] was used to generate a preliminary consensus sequence/structure for nt 1031–1250 based on energy minimization and structure conservation. The structure predicted without any experimental data is a multibranch loop with three hairpins, including one very thermodynamically stable long stem capped by a tetraloop (Fig 1A). When this model is compared to an alignment of 2515 unique influenza A sequences, base pairs are 91% conserved. When mutations occur, they preserve pairing via consistent (single point) and compensatory (double point) mutations (Fig 1).

### Secondary structure mapping of segment 5 1031–1250 (+)RNA structural motif

The consensus sequence of nts 1051–1171 (+)RNA of segment 5 was synthesized (M121) for *in vitro* structure mapping. The possibility of alternative folding of M121 was checked by using different annealing buffers. Native gel electrophoresis revealed that M121 folds into one conformation in wide range of Mg^2+^ concentration, from 5 mM to 25 mM, and also in [(Co(NH3)_6_]^3+^ at concentrations from 5 to 25 mM.

RNA secondary structure was mapped in a buffer containing 100 mM KCl, 5 mM MgCl_2_ at 23°C and pH 7. For enzymatic mapping RNase T1 (cleaves after unpaired G), RNase S1 (cleaves after unpaired nucleotide), and RNase V1 (cleaves after paired nucleotide) were used. Small molecules CMCT (modifies N3 of U and N1 of G when unpaired), DMS (methylates N1 of A and N3 of C when unpaired), and kethoxal (modifies G when unpaired) were used to probe the Watson–Crick face of bases. Lead ion cleavage and SHAPE mapping were used to identify flexible backbone regions.

**Fig 2:**
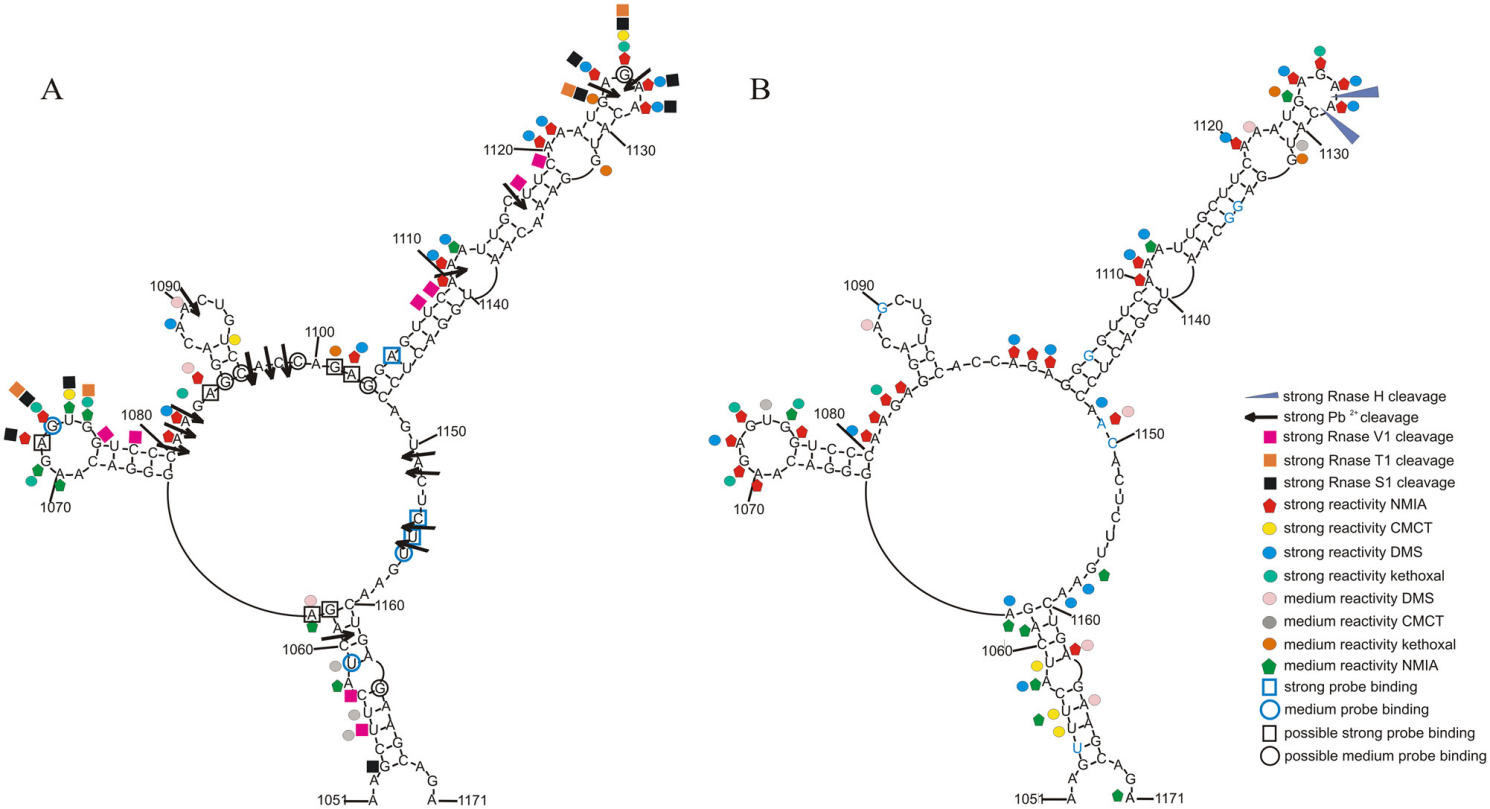
Secondary structure of influenza RNA motif. **A.** M121, isolated RNA with consensus sequence; secondary structure was predicted by RNAstructure 5.5 using chemical mapping results from SHAPE. **B.** Chemical mapping results of M121 in entire segment 5 (+)RNA (A/VietNam/1203/2004 (H5N1)); in blue is marked difference in sequence comparing to M121 on panel A.

**Fig 3:**
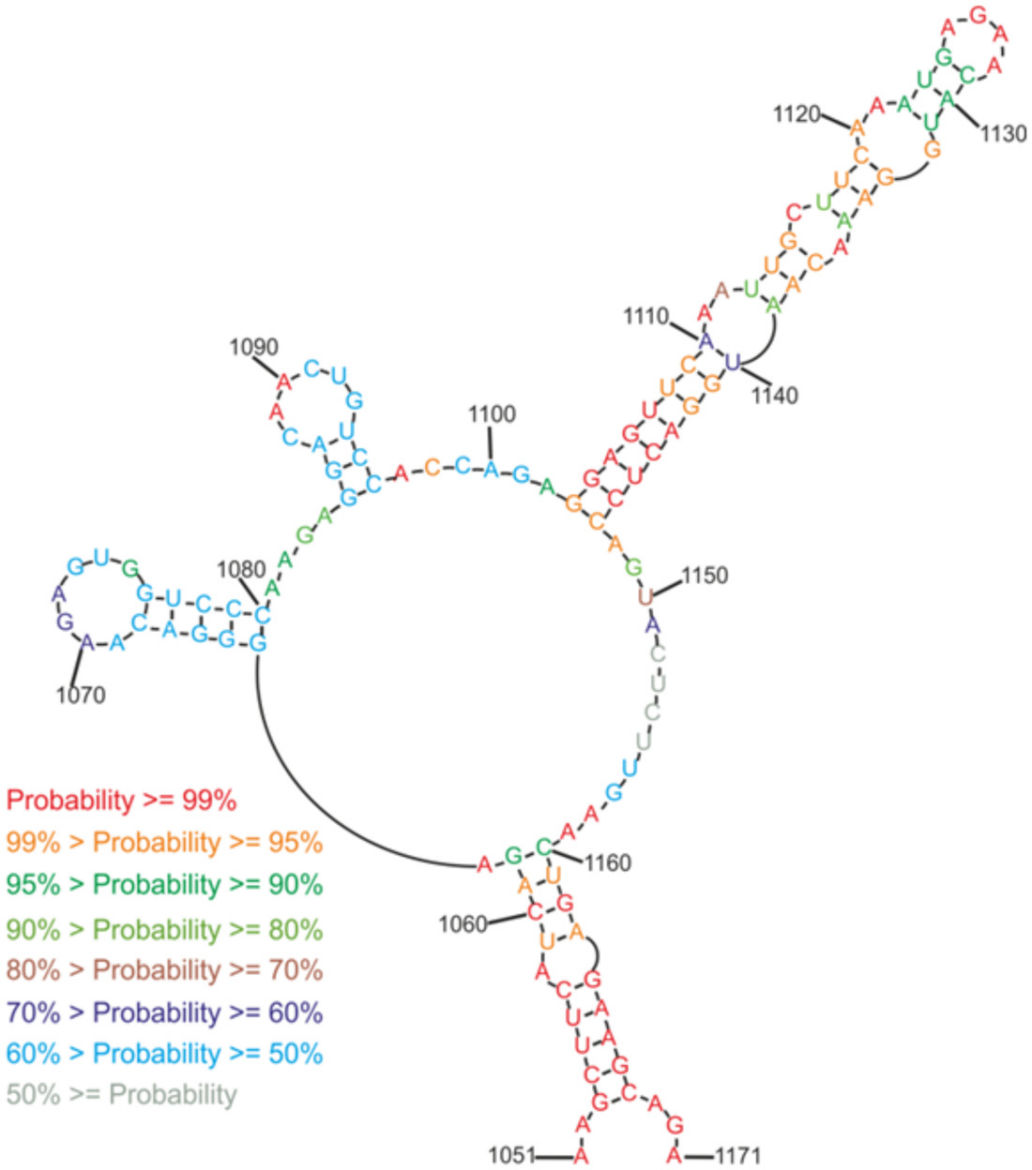
Predicted structure probability of M121 by RNAstructure 5.5 program.

The results of chemical mapping were used to constrain minimum free energy secondary structure prediction using RNAstructure v. 5.5 [33] (Fig 2A). The experimentally constrained structure is almost identical to the RNAalifold model; only one base pair (C1088-G1093; Fig 2A) is forced to be unpaired because of a strong CMCT hit at U1094. Otherwise, all chemical mapping data agree with the RNAalifold consensus model. Furthermore, incorporating experimental data into the calculation of the base pair partition function predicts the model pairs with a high calculated probability (Fig 3). Higher probability pairs are better-defined and correlate with an increased quality of prediction [34]. In particular nts 1102–1149, 1051–1063 and 1158–1171 are predicted with a very high level of confidence.

**Fig 4:**
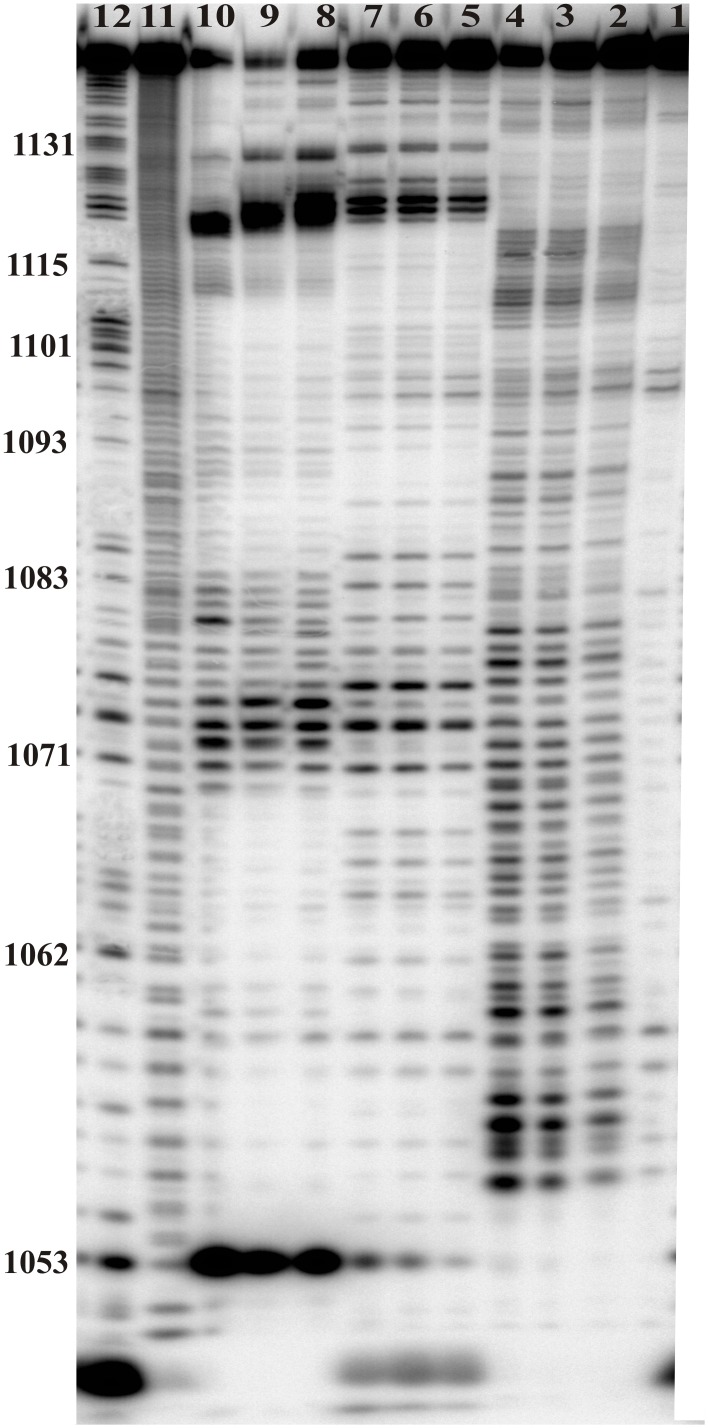
Enzymes mapping of M121. All reactions were conducted in 23°C for 30 min. Lane 1 –control reaction: M121 incubated in 100 mM KCl and 5 mM MgCl_2_, 10 mM Tris-HCl, pH 7, for 30 min in 23°C. Lanes 2–4—RNase V1 cuts in increasing concentration of enzyme: 0.5x10^-3^ U/μl, 1x10^-3^ U/μl and 3x10^-3^ U/μl, respectively. Lanes 5–7—RNase T1 cuts in increasing concentration of enzyme: 0.15 U/μl, 0.25 U/μl and 0.75 U/μl, respectively. Lanes 8–10—RNase S1 cuts in increasing concentration of enzyme: 0.05 U/μl, 0.3 U/μl, 1 U/μl, respectively, Lane 11—formamide ladder, Lane 12—RNase T1 ladder.

**Fig 5:**
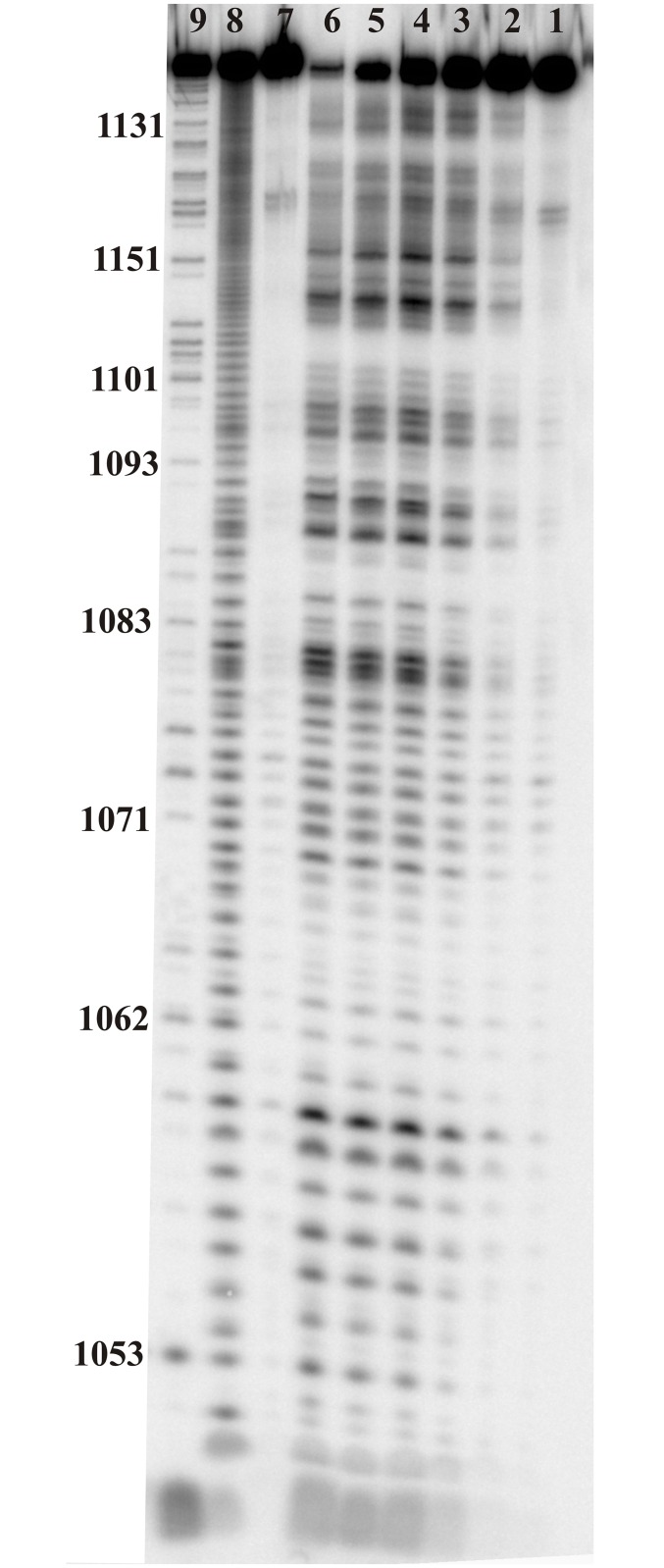
Lead ion cleavage of M121. RNA was incubated with 1 mM Pb(OAc)_2_, 100 mM KCl and 5 mM MgCl_2_, 10 mM Tris-HCl pH 7 in time course: lanes 1–6 - 0, 1, 5, 15, 30 and 60 min, respectively. Lane 7—control reaction: M121 incubated in 100 mM KCl and 5 mM MgCl_2_, 10 mM Tris-HCl pH 7 for 60 min. Lane 8—RNase T1 ladder. Lane 9—formamide ladder.

Chemical and enzymatic mapping are complementary to each other and show two the most flexible regions (Figs 2A, 4 and 5). Nucleotides 1070–1075 were the most reactive: modified by kethoxal, CMCT and NMIA and cut strongly by RNase T1 and S1—all in a hairpin loop (Fig 2A). The next, most reactive sites were nts 1124–1128, where DMS, kethoxal and NMIA hits were observed. Furthermore, Pb^2+^ cleavages were occurred after 1124 and 1126 nucleotides. There were RNase T1 cuts after G1124 and G1126 and S1 cuts after nucleotides 1124–1128 and no cleavage by RNase V1, which offers good support for this region being unpaired.

The predicted hairpin with looped nts 1087–1093 is slightly modified by chemicals: only A1089 and A1090 were reactive to DMS and U1094 to CMCT. Strong Pb^2+^ cleavage was observed after A1090 and there were no enzymes cleavages. Single stranded regions between hairpins undergo strong modification in 1081–1084 by NMIA, DMS and kethoxal. In this region strong Pb^2+^ cleavages were observed in region 1080–1082. This fragment is more accessible than hairpin 1085–1096. Lead ion cleavage also indicates additional flexible nucleotides: 1096–1098 and 1150, 1151 1154, 1155. These predicted single-stranded regions have only two identified chemical mapping hits (1101, 1102) as nts 1171–1132 were not able to be read-out by reverse transcription. Hairpins 1064–1080 and 1103–1147 and helix 1053-1062/1168-1160 are well confirmed in structural analysis. Additionally, mapped by NMIA, DMS and kethoxal nucleotides confirmed bulges: A1111-A1112, C1116, A1120-A1121, G1132, and A1058. After 1110 and 1116 nucleotides strong Pb^2+^ cleavages were observed, where 1110 is the strongest from all. (Figs 2A and 5). RNase V1 cuts indicate presence of helixes: 1053-1057/1168-1164, 1064-1068/1076-1080, 1103-1110/1140-1147 and 1117-1119/1133-1135. Small hairpin 1185–1096 and single-stranded multibranch-loop linker region between hairpins could have additional or alternative intramolecular interactions.

### Hybridization to Oligonucleotide Microarrays

Accessibility of M121 to oligonucleotide binding was studied using isoenergetic microarrays. Isoenergetic microarrays are built with short penta- and hexanucleotide probes which are 2’-O-methyl-RNA with locked nucleic acid (LNA) and 2,6-diaminopurine modifications at selected positions [22, 35]. In designing probes, the roughly-equal free energies of hybridization of probe/RNA duplexes was the goal [36–38]. Additionally, all chosen modifications increase the thermodynamic stability of hybridization duplex (probe/target RNA duplex), which allows the use of short probes. For example: 2,6-diaminopurine riboside (D) substituted for adenosine allows an additional hydrogen bond to form, making D-U pairs similar in stability to G-C [36]. Based on previous studies, LNAs in selected positions also increase the thermodynamic stability of duplexes. Careful design makes all probes used close to isoenergetic (in free energies of binding) [39]. In previous microarray mapping studies, primarily RNA secondary structure dictated the binding affinity of probes [22–24, 40].

The results of hybridizations of M121 to isoenergetic microarrays for three temperatures (4, 23 and 37°C) are collected in Table 2. The binding of modified probes are almost identical in 4°C and 23°C. Higher temperature eliminated binding of several probes, leaving only the most thermodynamically stable and specific. Probes which bind to target RNA were analyzed in terms of possible alternative binding sites. Predicted, other than target site, the possible binding sites (thermodynamically stable but not fully complementary; Table 2, last column) were used for identification of the unambiguous binding sites and possible accessible regions. All binding sites (the middle nt of the hybridized region for a probe) at 23°C were marked on the M121 secondary structure (Fig 2A). Accessible sites are in single stranded regions: 1073, 1154, 1155 and 1156. Binding site 1059 is in a weakly structured regions near bulge. Probe DC^L^UC^L^CG^L^ (symbol N^L^ indicate specific locked nucleic acid (LNA) nucleotide) has binding site at 1105 which is helix region. It is possible, because of G and A rich contest of target, that this probe form triplex instead of disrupting the double helix. Additionally, possible binding sites are in single stranded regions: 1084–1085, 1096, 1099, 1101–1103, 1126. Binding sites at 37°C provide target regions for potentially clinically useful antisense oligonucleotides.

### M121 motif in segment 5 (+)RNA of A/VietNam/1203/2004(H5N1)

The structure of M121 was checked in context of entire segment 5 (+)RNA sequence [A/VietNam/1203/2004(H5N1)]. The results of chemical mapping of nts 1051–1171 using NMIA, DMS, CMCT and kethoxal is presented on Fig 2B, where differences between this H5N1 sequence and the consensus sequence used for the *in vitro* construct are shown by blue letters. The binding site for the reverse transcription primer was chosen to be an appropriate distance to motif and enable to read out nts 1051–1171. In general, reactivity is in agreement with proposed secondary structure: e.g. hairpins 1064–1080 and 1103–1147 (Fig 2B). Also, medium RNase H cleavage after A1127 and A1128 in presence of DNA oligonucleotide TGTTCTC complementary to 1124–1130 show the accessibility of the hairpin loop (Fig 2B). The helix formed from nts 1053-1062/1168-1160 has several hits in the stem region: strong CMCT and medium NMIA modifications at 1056 and also reactivity at 1061 (NMIA) and 1163 (NMIA, DMS). The most noticeable difference between the truncated *in vitro* construct and the analogical region in the whole (+)RNA is at nts 1150–1156, which are unreactive in the (+)RNA. No alternative folds of the studied region (including pseudoknot) could explain these differences; they may be due to a tertiary interaction with another region in the (+)RNA, for example, the nearby region (nts 991–997) is complementary to 1150–1156.

### Inhibitory effect of oligonucleotides targeting the M121 motif in cell culture

Influenza RNAs were previously targeted with oligonucleotides as potential antiviral agents [41–43]. Several approaches using antisense oligonucleotides, siRNAs or miRNAs were published, where target regions were generally unique, well-conserved sequences, including genome packaging signals or start codons [31, 44–46]. Common used and good target were NP (segment 5) and the three polymerase RNAs (segment 1, 2, 3) [31, 47–50]. Oligonucleotide tools blocking or causing degradation of these segments not only disturb specific mRNAs but also block the accumulation of all viral RNAs, making them especially potent as influenza inhibitors. The conserved RNA structural motif presented in this current work contains possible new targets for oligonucleotides, that may complement those already found for the NP (+)RNA. Furthermore, here we use principles of oligonucleotide design that differ from previous studies.

**Fig 6:**
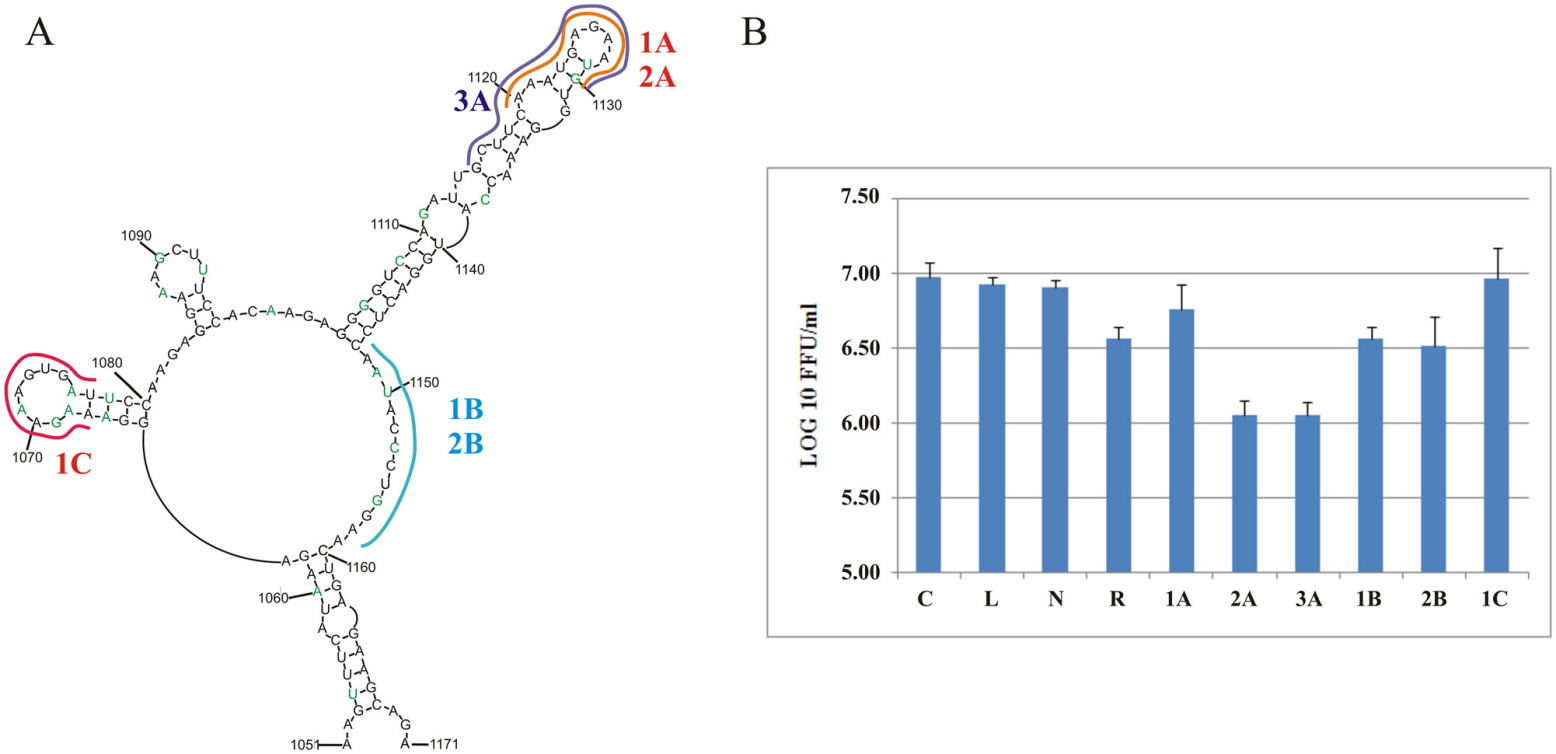
**A.** (+) RNA5 motif with marked complementary region to anisense oligonucleotides. In green were marked differences in sequence between A/California/04_NYICE_E3/2009 strain and consensus sequence of M121. **B.** Effect of antisense oligonucleotides targeting M121 motif of scIAV A/California/04_NYICE_E3/2009 in cell line MDCK-HA. C—control; L—control with lipofectamine, R—positive control with ribavirin; N—negative control with oligonucleotide N.

The importance of the M121 structural motif was investigated using an antisense oligonucleotide inhibition assay with a single-cycle infectious influenza A virus (sciIAV) [strain A/California/04_NYICE_E3/2009 (H1N1)] in MDCK-HA cells. The chosen strain differs slightly (82% sequence conservation, Fig 6) from the consensus sequence of M121: this was taken into account during oligonucleotides design to make them fully complementary. Oligonucleotide target sites were the two well-characterized hairpins at nts 1064–1080 and 1103–1147 and the single-stranded linker that possibly is involved in a tertiary interaction.

2’-O-Methylated oligonucleotides with LNA modifications: CAU^L^UCU^L^CAU^L^UU (2A) and CAU^L^UCU^L^CAU^L^UUGAAGC (3A) targeted the long hairpin and internal loop of M121 (region 1120–1130 and 1115–1130, respectively) (see the complementary regions, and strain sequence differences in Fig 6). These oligonucleotides were able to inhibit virus proliferation by a factor of 8.40 (0.93 LOG_10_ FFU/ml difference) (Fig 6). This hairpin thus appears to serve an important function in influenza A proliferation. Unmodified, 2’-O-methyl oligonucleotide CAUUCUCAUUU (1A) slightly inhibit virus: FFU/ml decrease by 1.64 times, which gives 0.16 LOG_10_ FFU/ml difference.

For (+)RNA5 of A/California/04_NYICE_E3/2009 the long distance interactions between regions: 1151-1157/991-997 is possible, but weaker: there are two mismatches (5’ACCCUGG/3’UGAGAAC). Targeting nts 1148–1158 with 2’-O-methyl oligonucleotides: 1B or 2B (UCCAGGGUAUU, UCC^L^AGG^L^GUA^L^UU), respectively, did not yield significant effects and only moderate inhibition for both 1B and 2B were observed. This is in agreement with *in vitro* mapping results, which show partial protection of the region from chemicals and lack of accessibility by microarray. Additionally, RNase H cleavage in presence of DNA oligonucleotide TCAAGAGTG complementary to 1150–1158 resulted in only weak cutting after U1155. It is likely that nts 1151–1157 is involved in weak tertiary interactions.

2’-O-Methyl oligonucleotide AUCACUUUCUU (1C) complementary to region 1067–1077 did not influence the virus replication, suggesting that this hairpin is less important to the virus or that it is less accessible to the oligonucleotide.

Presenting here approach leading from finding conserved secondary structure motifs in virus RNA to application of antisense oligonucleotides disturbing the motif possible function would be useful in therapeutics development.

## Conclusion

A bioinformatics analysis of the viral genome segment 5, which encodes nucleoprotein, revealed a conserved structural motif in the (+)RNA. The secondary structure proposed by energy minimization and comparative analysis agrees with structure predicted based on experimental data: using a 121 nucleotide *in vitro* RNA construct comprising an influenza A virus consensus sequence and also an entire segment 5 (+)RNA (strain A/VietNam/1203/2004 (H5N1)). The conserved motif consists of three hairpins with one being especially thermodynamically stable. The biological importance of this conserved secondary structure is supported in experiments using antisense oligonucleotides in cell line, which found that disruption of this motif led to inhibition of viral fitness. These results suggest that this conserved motif in the segment 5 (+)RNA might be a candidate for oligonucleotide-based antiviral therapy.
